# Microglial immune regulation by epigenetic reprogramming through histone H3K27 acetylation in neuroinflammation

**DOI:** 10.3389/fimmu.2023.1052925

**Published:** 2023-03-22

**Authors:** Minhong Huang, Emir Malovic, Alyssa Ealy, Huajun Jin, Vellareddy Anantharam, Arthi Kanthasamy, Anumantha G. Kanthasamy

**Affiliations:** ^1^ Parkinson Disorders Research Laboratory, Iowa Center for Advanced Neurotoxicology, Department of Biomedical Sciences, Iowa State University, Ames, IA, United States; ^2^ Center for Neurological Disease Research, Department of Physiology and Pharmacology, University of Georgia, Athens, GA, United States

**Keywords:** Histone acetylation, epigenetic reprogramming, microglial priming, trained immunity, neuroinflammation, neurodegenerative diseases

## Abstract

Epigenetic reprogramming is the ability of innate immune cells to form memories of environmental stimuli (priming), allowing for heightened responses to secondary stressors. Herein, we explored microglial epigenetic marks using the known inflammagen LPS as a memory priming trigger and Parkinsonian-linked environmental neurotoxic stressor manganese (Mn) as the secondary environmental trigger. To mimic physiological responses, the memory priming trigger LPS treatment was removed by triple-washing to allow the cells’ acute inflammatory response to reset back before applying the secondary insult. Our results show that after the secondary Mn insult, levels of key proinflammatory markers, including nitrite release, iNOS mRNA and protein expression, Il-6, Il-α and cytokines were exaggerated in LPS-primed microglia. Our paradigm implies primed microglia retain immune memory that can be reprogrammed to augment inflammatory response by secondary environmental stress. To ascertain the molecular underpinning of this neuroimmune memory, we further hypothesize that epigenetic reprogramming contributes to the retention of a heightened immune response. Interestingly, Mn-exposed, LPS-primed microglia showed enhanced deposition of H3K27ac and H3K4me3 along with H3K4me1. We further confirmed the results using a PD mouse model (MitoPark) and postmortem human PD brains, thereby adding clinical relevance to our findings. Co-treatment with the p300/H3K27ac inhibitor GNE-049 reduced p300 expression and H3K27ac deposition, decreased iNOS, and increased ARG1 and IRF4 levels. Lastly, since mitochondrial stress is a driver of environmentally linked Parkinson’s disease (PD) progression, we examined the effects of GNE-049 on primary trigger-induced mitochondrial stress. GNE-049 reduced mitochondrial superoxide, mitochondrial circularity and stress, and mitochondrial membrane depolarization, suggesting beneficial consequences of GNE-049 on mitochondrial function. Collectively, our findings demonstrate that proinflammatory primary triggers can shape microglial memory *via* the epigenetic mark H3K27ac and that inhibiting H3K27ac deposition can prevent primary trigger immune memory formation and attenuate subsequent secondary inflammatory responses.

## Introduction

Parkinson’s disease (PD) is a common debilitating neurodegenerative disease placing a heavy socioeconomic burden on the healthcare system all over the world. The exact cause of this disease remains unknown, but it may be attributed to the entanglement of genetic, epigenetic, and environmental factors. Persistent neuroinflammation primarily mediated by microglia, the principal innate immune cells of the brain, is a pathophysiological hallmark of PD. As a specialized population of phagocytic cells in the central nervous system (CNS), microglia are capable of sensing environmental neurotoxicants and inflammatory signaling factors from outside the CNS. In this regard, risk factors from the surrounding environment can induce microglia to release an excess of inflammatory cytokines, generating exaggerated neuroinflammation, and consequently impacting the progression of PD in later life (1). Recently, an intriguing concept has emerged that such insult-induced microglia can also shape immune-response memories through epigenetic reprogramming, termed trained immunity (2, 3). In the context of environmentally linked PD, trained immunity refers to the ability of innate immune cells to form memories of environmental inflammatory stimuli that enable them to mount a heightened response to a secondary inflammatory insult *via* epigenetic marks (3–5). Epigenetic reprogramming represents the deposition of epigenetic modifications on the chromatin as epigenetic marks. Among epigenetic marks, key histone marks include histone 3 lysine 27 acetylation (H3K27ac) marks at distal enhancers (along with histone 3 lysine 4 monomethylation (H3K4me1) and histone 3 lysine 4 trimethylation (H3K4me3) marks at the promoters of stimulated genes (6). Once the initial stimulus is removed, microglia return to their steady state but with a mark or marks on the histone tail, thus becoming trained microglia (7, 8). This training process records the environmental inflammatory insult in the long-lasting memory of innate immunity (3, 7). This endows microglia in the brain with the ability to rapidly respond and adapt to environmental challenges, but this adaptive characteristic does not always turn out to be beneficial to the brain. Overly reactive microglia in misguided trained immunity can create exaggerated inflammatory responses to recurring stimuli that keep them in a chronic hyperinflammatory state. Because of this long-lasting inflammation, together with transcriptional expression perturbation by epigenetic reprogramming during the process, misguided trained immunity is thus thought to be an important contributor to PD progression. However, the molecular mechanism underlying this emerging concept is far from clear.

Environmental inflammatory stimuli have been shown to provoke neuroinflammatory responses. As an environmental inflammatory stimulus, chronic manganese (Mn) exposure has been linked to Parkinsonism in humans (9). Excessive Mn accumulates in the basal ganglia of the brain resulting in neuronal dysfunction, particularly in the globus pallidus, subthalamic nucleus, striatum and substantia nigra, where extrapyramidal motor functions are controlled (10). Mn-induced Parkinsonism (also known as manganism) resembles PD in many ways, both of whose main symptoms include resting tremors, bradykinesia, muscular rigidity, postural instability, and several non-motor symptoms (11). Despite similarities, significant differences in pathological changes and pharmacological responses do exist between PD and Mn neurotoxicity.

Similar to trained immunity, microglial priming is the process of making the microglia much more susceptible to a secondary inflammatory stimulus, which subsequently triggers a heightened inflammatory response. Recently, we have shown that exposure to the neurotoxicant Mn potentiates the NLRP3-dependent inflammasome inflammatory response in primed microglia (12). A long-held assumption is that curbing the environmental exposure would be an efficient remedy against neurotoxic poisoning. However, emerging evidence implies that immune memory of previous exposure to inflammatory conditions could render subjects susceptible to a secondary inflammatory exposure (2, 3, 13). Indeed, features of trained immunity reportedly overlap with microglial priming (3, 6, 7, 14). Thus, we investigated the concept of trained immunity using LPS-primed treatment paradigms. Specifically, we studied microglia-mediated epigenetic marks by using LPS as a memory priming trigger and Mn as the secondary environmental insult. To mimic the immune memory events, we removed the memory priming trigger LPS, allowed microglia a certain amount of time to return to a relatively steady state, and then characterized the epigenetic mark-associated alterations following exposure to the secondary environmental trigger Mn. If trained immunity shares similarities with microglial priming in our treatment paradigm, we hypothesized that (1) the primary memory trigger LPS deposits epigenetic marks *via* key histone modification; (2) Mn exposure recalls the memory of the primary trigger, leading to an exaggerated inflammatory response; and (3) inhibiting the deposition of histone modification marks facilitates the removal of memory formation, resulting in reduced inflammatory signaling. We examined this epigenetic reprogramming-shaped, microglial memory-evoked inflammatory response using a mouse microglial cell (MMC) line, primary mouse microglia (PMG), the MitoPark PD mouse model, and postmortem human PD brains. With a focus on H3K27ac, we also used the p300/CBP pharmacological inhibitor GNE-049 to suppress epigenetic memory-shaping in MMC, and primarily examined the H3K27ac-regulated, inflammation-associated transcription factor *Nos2* (iNOS). Herein, we provide evidence on how primary triggers shape microglial memory *via* epigenetic marks that can recognize subsequent secondary inflammatory insults.

## Results

### Inflammatory responses in trained immunity resemble microglial priming

**Figure 1:**
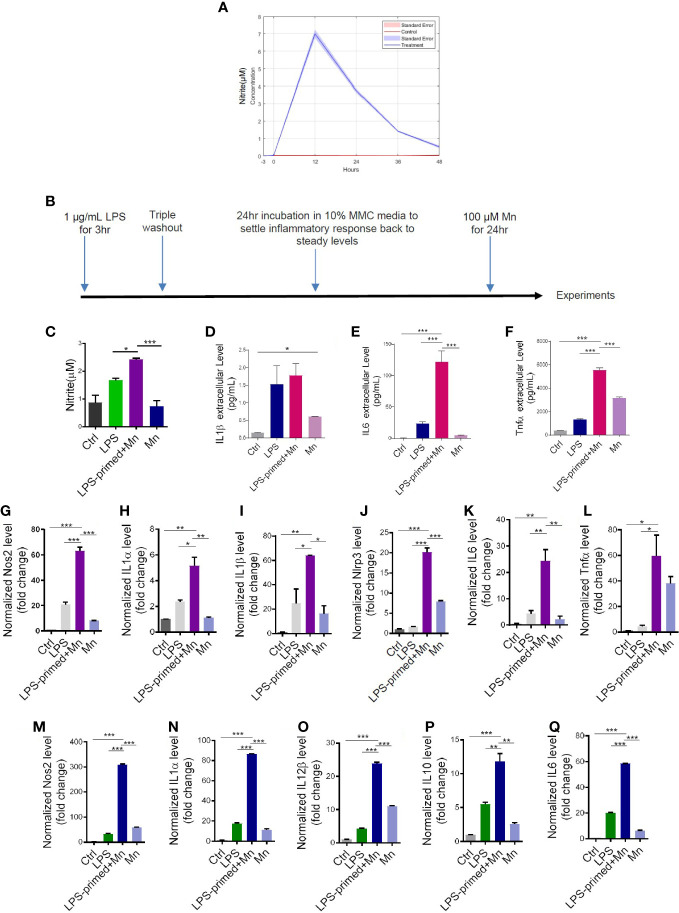
Heightened inflammatory responses in trained immunity. **(A)** LPS-induced time response experiment in mouse microglial cells (MMCs) *via* Griess assay. Data are means ± SEM of two individual experiments performed in four replicates. Light blue area denotes SEM. **(C)** Nitric oxide release from LPS- and Mn-treated, primed MMC *via* Griess assay (n=8-9), following the treatment paradigm in **(B)**. LPS+Mn denotes the treatment with LPS for 3 h, a 24-hour recovery, and then Mn for 24 h. **(D–F)** Luminex bead-based cytokine assays (n=3) of pro-inflammatory cytokine release following the treatment paradigm in **(B)**. **(G–L)** RT-qPCR analysis of *Nos2*, *IL-1α*, *IL-1β*, *NLRP3*, *IL-6*, and *Tnfα* in Mn-exposed, LPS-primed PMG cells following the treatment paradigm in **(B)**. Two individual experiments (n=2) were performed. **(M–Q)** RT-qPCR analysis of *Nos2*, *IL-1α*, *IL-12β*, *IL-10*, and *IL-6* in trained MMC, using the treatment paradigm in **(B)**. Two individual experiments (n=2) were performed. Data show mean ± SEM of one-way ANOVA followed by Tukey’s *post hoc* test. Ctrl, control; *p ≤ 0.05; **p<0.01; ***p<0.001.

Our prior findings show that exposing microglial cells to the neurotoxicant Mn immediately following LPS priming (1 μg/mL for 3 h) activated the NLRP3 inflammasome inflammatory signaling pathway (8). To determine the optimal time of LPS exposure and to establish the neuroimmune paradigm in the current study, we first performed a time-course assessment of the inflammatory response following LPS priming using iNOS activity by Griess assay. LPS-primed MMC cells induced a maximal proinflammatory response at 12 h and gradually recovered to a near steady-state after 48 h of LPS priming (**Figure 1A**). Thus, we adopted this approach as our priming treatment paradigm, that is, LPS as a memory priming trigger and Mn as the secondary environmental insult after a 48-h interval that encompasses a triple wash and steady-state recovery (**Figure 1B**). A pilot RT-qPCR experiment with 48-h recovery time in PMG cells revealed a dramatic increase of IL-1β and iNOS following Mn exposure (**Figure S1**), similar to the heightened secondary inflammatory response of microglial priming without recovery time. Note too that, although not statistically significant, the mean transcription levels of IL-1β and iNOS in LPS-primed only cells did not numerically return to the baseline during the recovery phase after LPS triple-washout (**Figures S1A, B**), thus providing evidence that microglial cells retain a heightened state after exposure to primary memory trigger. These results suggest that LPS-primed microglia can form microglial memory and become more sensitive to a secondary stimulus. Because the inflammatory responses can be possibly confounded by reduced cell viability due to a prolonged Mn exposure when extending the treatment paradigm, the recovery time of 24 h was used in our treatment paradigm (**Figure 1B**).

Next, we evaluated whether immune-regulated memory occurs as expected and how it affects neuroinflammation. Griess assay shows that Mn, as a secondary stimulus, induced an exaggerated inflammatory response in LPS-primed, 24-h-recovered MMC cells (**Figure 1C**). Cytokine assay confirms that Mn significantly potentiated the secreted levels of IL-6 and TNFα in primed MMCs (**Figures 1D–F**). Our RT-qPCR of LPS-primed PMG and MMC lysates shows Mn also exaggerated the inflammatory responses of *Nos2*, *IL-1α*, *IL-1β*, *Nlrp3*, *IL-6*, and *Tnfα* (**Figures 1G–L**) and *Nos2*, *IL-1α*, *IL-12β*, *IL-10*, and *IL-6* (**Figures 1M–Q**), respectively. We also explored the possibility of using Mn as the primary memory trigger followed by LPS/Mn treatment. RT-qPCR (**Figure S2A**) and Griess (**Figure S2B**) assays showed that Mn priming can induce trained immunity with LPS being a more potent second insult as compared to Mn. Collectively, our LPS-trained immunity treatment paradigm shows that Mn exposure recalled the memory of the primary memory trigger, leading to an exaggerated inflammatory response.

### Microglial immune regulation by epigenetic reprogramming

**Figure 2:**
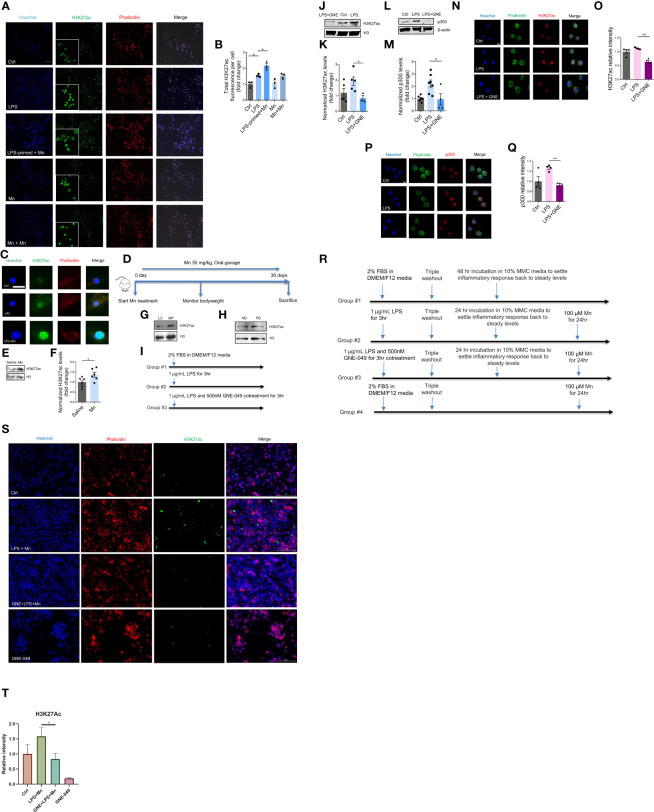
H3K27ac deposition in microglial cells and mice in response to LPS priming and subsequent Mn exposure. **(A)** Representative ICC images of H3K27ac in MMCs primed with LPS and treated as indicated, with phalloidin-stained cytoplasm in red, Hoechst-stained nuclei in blue, and H3K27ac in green (n=3). Scale bar, 100 µm. **(B)** Immunofluorescence microscopy analyses of total H3K27ac intensity per cell. Calculation was automatically performed by Keyence microscope software. **(C)** Immunofluorescence microscopy analysis of H3K27ac in Mn-exposed, LPS-primed PMG cells. A single PMG cell is shown because of their low cell density resulting from their reduced viability during prolonged treatments. Scale bar, 10 µm. **(D)** Representative diagram of Mn treatment of C57BL/6J mice (n=6). **(E)** Representative Western blot and **(F)** densitometry assessing H3K27ac deposition in the striatum of Mn-exposed C57BL/6J mice (n=6), as a measure of Mn potential to promote H3K27ac deposition *in vivo*. Representative Western blots of increased H3K27ac deposition in the substantia nigra of **(G)** MitoParks and **(H)** postmortem human PD brains. ND, non-disease subjects; PD, PD patients. **(I)** Representative diagram of MMC treatment paradigm using GNE-049 to suppress memory formation by inhibiting LPS-induced deposition of epigenetic marks **(**results depicted in **J–Q)**. **(J)** Representative Western blot and **(K)** densitometry assessing H3K27ac deposition in LPS treatment or LPS and GNE-049 co-treatment (n=3~5). GNE denotes GNE-049. **(L, M)** Representative Western blot and densitometry assessing of p300 deposition in LPS treatment and LPS with GNE-049 co-treatment (n=4~7). Immunofluorescence microscopy analyses of **(N, O)** H3K27ac and **(P, Q)** p300 in MMCs with treatments as indicated (n=4). Scale bar, 5 µm. **(R)** Representative diagram of MMC treatment paradigm to examine the effects of GNE-049 on H3K27ac upon a secondary Mn insult **(S, T)**. **(S, T)** Representative immunofluorescence microscopy analysis of H3K27ac in Mn-exposed, LPS-primed MMCs cotreated with GNE-049 inhibitor. Scale bar, 100 µm. Data show mean ± SEM of one-way ANOVA followed by Tukey’s *post hoc* test. Ctrl, control; *p ≤ 0.05; **p<0.01.

Although the mechanisms underlying microglial priming and trained immunity have not yet been well defined, emerging evidence implies that immune regulation of trained immunity is deposited through epigenetic reprogramming and that microglial priming in the brain can be viewed as trained immunity (3, 7). It is well known that epigenetic reprogramming involving histone modification positively and negatively affects gene expression. Accumulating studies suggest that immune regulation-associated memory formed by environmental stimuli is stored by the specific deposition of H3K4me1, H3K4me3, and H3K27ac (7, 15). But whether Mn activates microglial immune regulation through deposition of epigenetic markers and the identity of which epigenetic marker(s) store(s) long-term microglial immune-regulated memory of their previous encounters are unknown. Our immunocytochemistry (ICC) analysis demonstrates increased H3K27ac accumulation per cell in Mn-exposed, LPS-primed MMCs (**Figure 2A**), and LPS- alone group induced increased H3K27ac deposition even higher in Mn exposure. Note that Mn treatment enlarged MMC cell bodies as is expected of activated microglia, thus making LPS-primed, Mn-treated MMCs appear slightly less intensely green than the less swollen LPS-trained MMCs (16, 17). Based on total H3K27ac intensity per cell, the LPS+Mn treatment induced more K27ac than LPS-alone per cell (**Figure 2B**), whose results were confirmed in PMGs (**Figure 2C**). We also investigated the effect of utilizing Mn as both the primary memory trigger and the secondary stimulus on H3K27ac deposition. Repeated Mn exposure also increased H3K27ac accumulation per cell but the effect was not significant (**Figures 2A, B**). To extend our findings, an *in vivo* study was performed, in which C57BL/6J mice were administered 100 mg/kg Mn by oral gavage for 30 days (**Figure 2D**). Western blot analysis shows increased deposition of H3K27ac in Mn-treated mouse striatal tissues when compared to saline-treated controls (**Figures 2E, F**). The MitoPark mouse model recapitulates the progressive nature and key pathophysiological features of PD, including most importantly microglial inflammation (18). According to our previous data (19), H3K27ac accumulation in substantia nigral tissues collected from both MitoPark mice and postmortem human PD brains was greater than that from control brains in Western blots (**Figures 2G, H**). Collectively, these findings suggest an important role and clinical relevance of H3K27ac in PD.

To study whether LPS induces priming-associated H3K27ac through p300, we used the p300/CBP pharmacological inhibitor GNE-049. Recent publications report that GNE-049, as a promising small-molecule therapy. Targets p300 and blocks H3K27ac at enhancers to efficiently suppress the growth of castration-resistant prostate cancers and hematological malignancies (20, 21). To reduce H3K27ac levels, MMC cells were co-treated with 1 µg/mL LPS and 500 nM GNE-049 for 3 h (**Figure 2I**). Compared to the LPS-priming- alone group, levels of H3K27ac and p300 decreased in the GNE-049/LPS co-treatment group as revealed by immunoblotting (**Figures 2J–M**, respectively) and ICC images (**Figures 2N–Q**, respectively).

Next, we investigated whether inhibiting p300/CBP by GNE-049 prevents Mn-potentiated H3K27ac deposition from LPS-primed microglial cells. MMCs were treated with 1 µg/mL LPS in the presence and absence of 500 nM GNE-049 for 3 h. The cells were then triple- washed, incubated 24 h to recover to normal state and treated with 100 µM Mn for another 24 h (**Figure 2R**). ICC analysis consistently demonstrates an exaggerated level of H3K27ac by Mn treatment in LPS-primed MMCs, whereas co-treatment with GNE-049 attenuated this potentiation effect (**Figures 2S, T**). Of note, GNE-049 treatment alone had no effect on H3K27ac levels. These results collectively indicate that H3K27ac possibly plays an important role in immune regulation.

### Inhibiting deposition of epigenetic mark H3K27ac through p300 suppresses iNOS and its pathway-associated inflammatory factors

**Figure 3:**
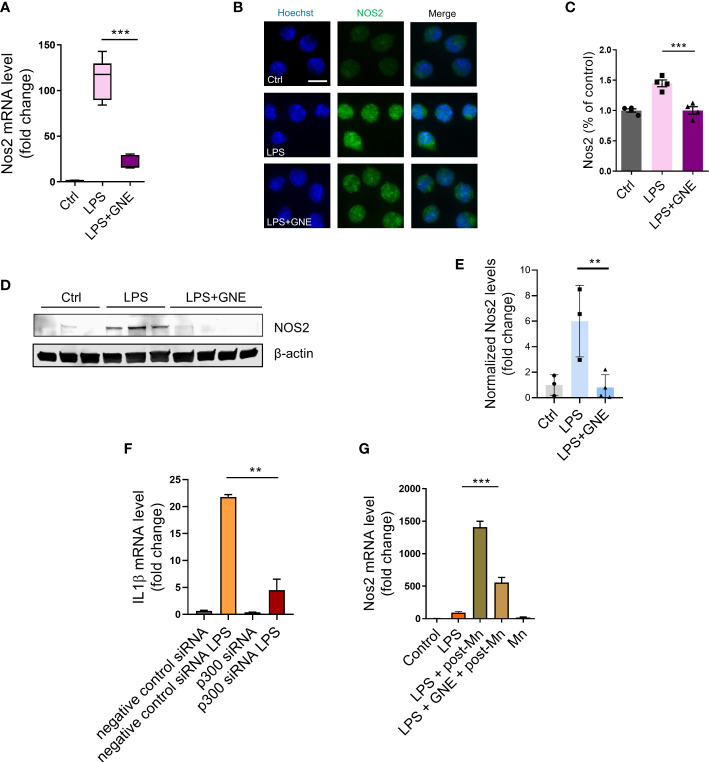
Inhibiting deposition of epigenetic mark H3K27ac through p300 suppresses iNOS signaling. **(A)** RT-qPCR analysis of *Nos2* in primed MMC, indicating that the inhibitor GNE-049 decreased transcriptional expression of *Nos2* by inhibiting p300/CBP. Two individual experiments (n=3) were performed. **(B, C)** Immunofluorescence microscopy analyses of *Nos2* in GNE-049 co-treated, primed MMCs (n=4). Scale bar, 10 µm. Immunoblotting analyses of **(D, E)** *Nos2* (n=3). **(F)** RT-qPCR analysis demonstrated that the relative expression levels of *IL-1β* in p300 knockdown (KD) following LPS treatment, compared to MMC with negative control siRNA. **(G)** RT-qPCR analysis of *Nos2* in primed MMC (n=4~12) following the treatment paradigm to examine the effects of GNE-049 on the transcription levels of *Nos2* upon a secondary Mn insult. Data show mean ± SEM of one-way ANOVA followed by Tukey’s *post hoc* test. Ctrl, control; **p<0.01; ***p<0.001.

Our data above identified that H3K27ac deposition serves as a primary trigger-induced, long-lived memory and regulates inflammatory responses. Our next step was to explore the downstream molecular mechanism underlying H3K27ac-mediated immune regulation. For this, RT-qPCR, ICC, and immunoblotting were performed to check multiple neuroinflammation-associated factors. GNE-049 co-treatment decreased iNOS transcript levels in RT-qPCR (**Figure 3A**) as well as its protein levels in ICC images (**Figures 3B, C**) and Western blot (**Figures 3D, E**).

Next, we knocked down p300 in MMC cell line to preliminarily confirm that LPS induces iNOS pathway-associated inflammatory factors through H3K27ac, whose results showed the decreased transcription expression of *IL1β*, *Nos2*, *Nlrp3*, and *Tnfα* (**Figure 3F**; **Figures S3A–C**). Western blot also showed that the translation levels of p300, iNOS, and H3K27ac decreased (**Figure S3D**). Then we explored how GNE-049 protected microglia from a secondary Mn insult following a 24-h steady-state recovery interval. Thus, after the 3-h LPS primary stimulus, a triple washout, and a 24-h recovery time to return the inflammatory response to steady-state levels, we introduced a 24-h Mn (100 μM) insult. Interestingly, GNE-049 co-treatment significantly reduced iNOS expression in the Mn-exposed, LPS-primed MMCs (**Figure 3G**).

**Figure 4:**
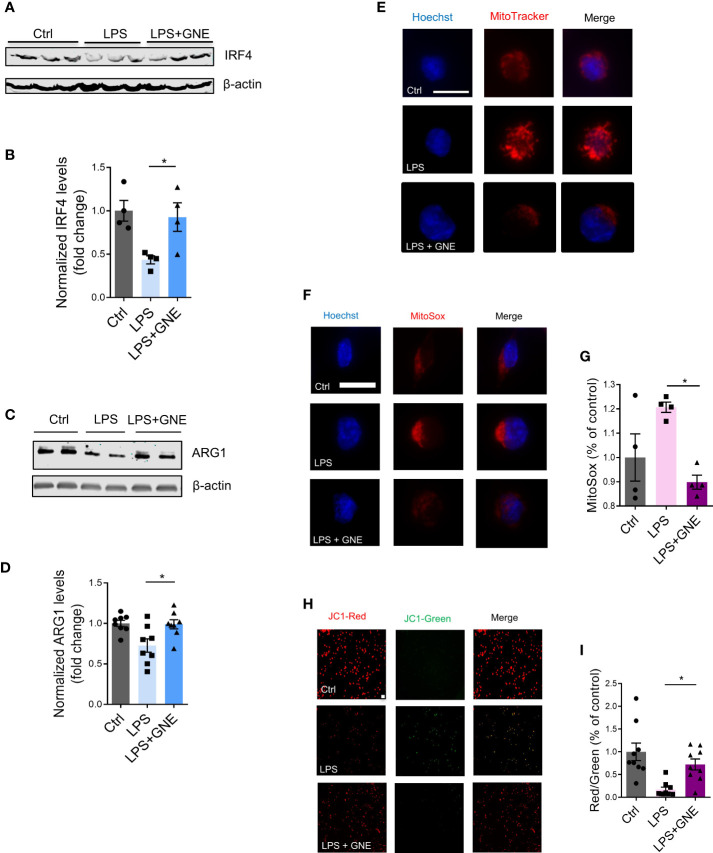
GNE-049 increases the expression of the M2 polarization marks IRF4 and ARG1. Representative Western blots and densitometry assessing **(A, B)** IRF4 (n=4) and **(C, D)** ARG1 (n=8) in MMCs. **(E)** MitoTracker probe staining shows changes in mitochondrial morphology in MMCs cotreated with GNE-049 (n=3). Scale bar, 10 µm. **(F, G)** MitoSOX assays reveal that GNE-049 reduced LPS-induced superoxide generation by inhibiting H3K27ac deposition (n=4). Scale bar, 10 µm. **(H)** JC1 probe (scale bar, 50 µm) and **(I)** quantitative analysis of Δψm by the ratio of red and green fluorescence. Data show mean ± SEM of one-way ANOVA followed by Tukey’s *post hoc* test. Ctrl, control; *p ≤ 0.05.

The dynamic changes of M1/M2 microglial phenotypes are critically involved in neuroinflammation, in which M2 polarization is associated with neuroprotective functions (22, 23). To check whether GNE-049 promotes M2 polarization, we tested M2 markers in GNE-049-treated microglial cells. GNE-049 co-treatment increased the expression of the M2 polarization marks IRF4 (**Figures 4A, B**) and ARG1 (**Figures 4C, D**).

For our treatment paradigm, we also briefly checked NLRP3 inflammasome activation in MMCs. Similar to microglial priming, LPS treatment increased NLRP3 levels, while GNE-049/LPS co-treatment almost abolished its expression (**Figure S4A**). GM-CSF has been reported as a trained immunity modulator involved in enhancing inflammatory responses (6, 24). GM-CSF expression was increased by LPS, but reduced by co-treating with GNE-049, suggesting the beneficial effects of GNE-049 on inflammatory responses to trained immunity (**Figures S4B, C**).

Mitochondrial stress is a key pathophysiological process of environmentally linked PD. Crosstalk has been reported between mitochondrial stress and inflammation (25, 26). In addition, we recently reported that mitochondrial dysfunction leads to epigenetic dysregulation in environmentally linked PD (19). As such, we examined the effects of GNE-049 on memory trigger-induced mitochondrial stress. MMCs co-treated with GNE-049/LPS displayed significantly decreased mitochondrial circularity *via* MitoTracker staining (**Figure 4E**), while MitoROS production markedly decreased as determined *via* MitoSox assay (**Figures 4F, G**). Moreover, the mitochondrial membrane potential increased in GNE-049 co-treated MMCs as revealed by an increased red/green ratio in the JC-1 assay (**Figures 4H, I**). These findings suggest that GNE-049 protects microglia from primary trigger-induced structural and functional derangement of mitochondria. Therefore, besides suppressing iNOS and NLRP3 factors, GNE-049 can also potentially protect microglial cells from inflammation by alleviating mitochondrial stress.

To sum up, blocking deposition of the epigenetic mark H3K27ac inhibited iNOS and its associated inflammatory factors. GNE-049 has beneficial pharmacological effects against LPS induced inflammation. In our studies, it inhibited the H3K27ac deposition that forms initial memory in microglia and consequently suppressed iNOS and NLRP3- associated inflammation.

### Other epigenetic marks H3K4me3 and H3K4me1 in immune memory regulation

**Figure 5:**
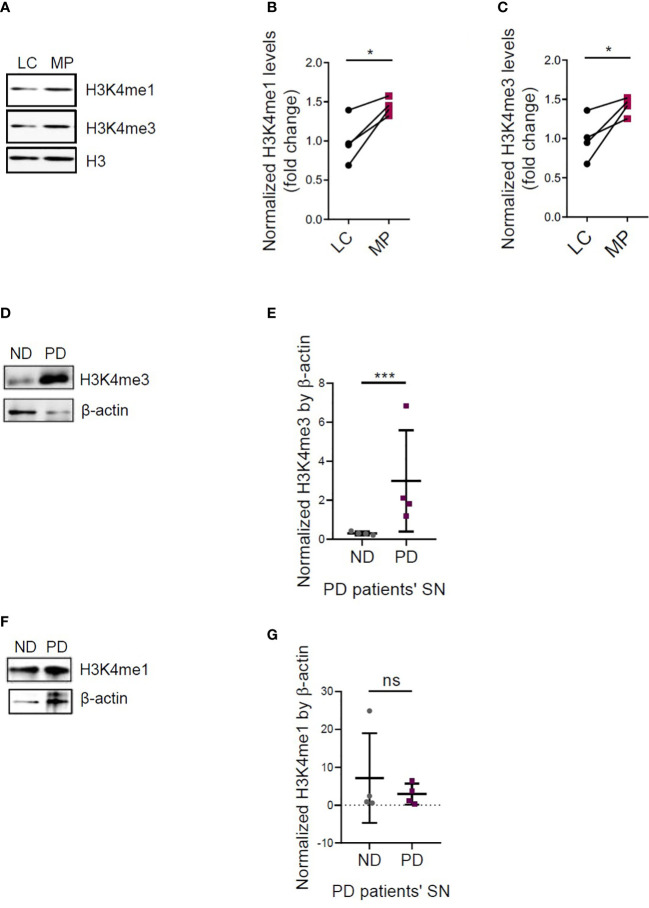
Increased H3K4me3 and H3K4me1 deposition in MitoPark mice and postmortem human PD brains. **(A)** Representative Western blots and **(B, C)** densitometry assessing H3K4me1 and H3K4me3 deposition in the substantia nigra (SN) of MitoPark mice (n=4). Representative Western blots and densitometry assessing **(D, E)** H3K4me3 and **(F, G)** H3K4me1 deposition in the SN of postmortem human PD brains (n=4). LC, littermate controls; MP, MitoParks; PD, human PD patients; ND, non-disease subjects. Data show mean ± SEM of one-way ANOVA followed by Tukey’s *post hoc* test. Ctrl, control; ns, not significant; *p ≤ 0.05; ***p<0.001.

Furthermore, we examined other trained immunity marks, that is, H3K4me3 and H3K4me1. Immunoreactivities of H3K4me1 and H3K4me3 increased in Mn-treated, LPS-primed PMGs, compared to LPS-primed alone cells, as displayed by ICC (**Figure S5**). Then we used MitoParks to further explore the relevance of H3K4me3 and H3K4me1 with PD. We examined both the substantia nigra and olfactory bulb to study the regional distribution of H3K4me3 and H3K4me1, since olfactory dysfunction is one of the premotor symptoms reflecting early deposition of Lewy pathology in PD patients (27, 28). As confirmed by immunoblotting, H3K4me3 and H3K4me1 increased significantly in substantia nigral tissues of 16~20-week MitoParks relative to littermate controls (**Figures 5A–C**), while no significant change occurred in the olfactory bulb (**Figure S6**). Next, regarding clinical relevance, H3K4me3 deposition increased significantly in the substantia nigra from postmortem human PD brains (n=4) when compared to age-matched healthy controls in Western blots of whole cells lysates (**Figures 5D, E**). The slightly higher mean signal density for H3K4me1 in postmortem human PD brain samples was not significant (**Figures 5F, G**). These results collectively indicate that H3K4me3 along with H3K4me1 is potentially an epigenetic mark for immune regulation.

## Materials and methods

### Chemicals and reagents

Fetal bovine serum (FBS), RPMI, L-glutamine, penicillin, streptomycin, MitoTracker Red, MitoSOX Red stains, IRDye-tagged secondary antibodies, Hoechst nuclear stain, and other cell culture reagents were purchased from Invitrogen (Gaithersburg, MD). LPS (*Escherichia coli* 0111: B4, endotoxin content 6.6000000 EU/mg) and the Bradford protein assay kit were ordered from MilliporeSigma (Burlington, MA). The p300/H3K27ac inhibitor GNE-049 was purchased from MedChemExpress (Monmouth Junction, NJ). p300 siRNA was purchased from MilliporeSigma (Burlington, MA) for transfection (Cat. #EMU078861-50UG). The phalloidin antibody was purchased from Thermo Fisher Scientific, acetylation inhibitor cocktail from Santa Cruz, and protease and phosphatase inhibitor cocktail from Life Technologies.

### Cell cultures and treatment paradigms

The immortalized mouse microglial cell line (MMC) was donated by D.T. Golenbock (University of Massachusetts Medical School, Worcester, Massachusetts, USA) and characterized by our group (29). MMC cells were grown in Dulbecco’s modified eagle medium/nutrient mixture F-12 (DMEM/F-12) supplemented with 10% FBS, 1% sodium pyruvate, 1% glutamine, and 1% penicillin-streptomycin. Cell treatments were completed in 2% FBS-containing DMEM/F-12 medium. For the LPS time-response experiment (**Figure 1A**), time points include 0, 12, 24, 36, and 48 h.

For LPS- and Mn-primed treatments, the cells were treated with 1 µg/mL LPS or 100 µM Mn and incubated for 3 h. After the 3-h interval, the cells were triple-washed with full serum medium to completely remove the LPS/Mn. We then added 10% FBS-containing DMEM/F-12 medium to the cells, and they were incubated 24 h to allow the cells to recover to their normal state. Finally, 100 µM Mn or 1 µg/mL LPS was applied to the cells for another 24 h. After the final incubation period, cells were either collected for RT-qPCR or Western blot or applied to later processes.

For LPS/GNE-049 co-treatment experiments (**Figures 2E–M**, **4**, **5**, and **S5A**), MMCs were treated with 1 µg/mL LPS in the presence or absence of 500 nM GNE-049 for 3 h. Cells were washed to completely remove LPS and then harvested either immediately for experiments or given 48 h to recover to a steady state.

Some MMC flasks were applied to another treatment paradigm. Either 1 µg/mL LPS only or 1 µg/mL LPS and 500 nM GNE-049 was/were added to MMC cells for 3 h. After a triple-wash to completely remove LPS and GNE-049 and a 24-h incubation in 10% FBS-containing DMEM/F-12 medium, 100 µM Mn was applied to the cells for 24 h.

### Primary mouse microglia isolation

We followed the standard protocol of primary microglia isolation previously developed by Sarkar et al. (30). In brief, mixed glial cultures from P0 C57BL/6J mouse pups were obtained. Primary mouse microglia were isolated from mixed glial cultures by using magnetic beads and were maintained in similar media as used for MMC, except that 1% nonessential amino acids were added.

### Multiple cytokine Luminex immunoassays

Cytokine levels were assessed *via* Luminex assays following the protocol previously utilized in our lab. Over a short interval, either PMG or MMC cells were treated in 96-well plates with 100–900 μL of 2% FBS-containing DMEM-F12 medium. After treatment, 40 μL of treatment medium was collected, and 40 μL of primary antibody conjugated to magnetic microspheres were added and incubated overnight at 4°C in a clear-bottom, black 96-well plate. Following this incubation, each well was triple-washed in a magnetic washer. The plate was then incubated for 1 h with the desired secondary antibodies. The samples then had streptavidin/phycoerythrin added to them and were incubated for another 30 min. Finally, a Bio-Plex reader was used to read the 96-well plates. A standard curve of all the cytokines was prepared using standard cytokines.

### Animal studies

A 12-h light cycle in a climate-controlled mouse facility (22 ± 1°C) with food and water available *ad libitum* was used for all mice bred and maintained. MitoPark transgenic mice were a kind gift from Dr. Nils-Goran Larsson and were originally generated in his laboratory at the Max Planck Institute for Biology of Ageing by conditionally knocking out TFAM through control of the dopamine transporter (DAT) as previously described (31). All MitoPark mice used for this study were bred from the MitoPark breeding colony at Iowa State University (ISU). Equal numbers of male and female MitoParks were assigned to experiment groups comprising 16- to 20-week-old, age-matched C57BL/6 mice (TFAM^+/LoxP^: Dat^+/+^ from MitoPark litters or parental-strain litters) or MitoPark mice (Dat^+/Cre^: TFAM^LoxP/LoxP^), which were genotyped to confirm their identity and further characterized using VersaMax for monitoring locomotor activity and RotaRod for testing coordination of movement. For Mn animal studies, after acclimating for 3 days, C57BL/6 mice were orally gavaged daily with 30 mg/kg of MnCl_2_ for 30 days, after which mice were sacrificed. Investigators involved with data collection and analysis were not blinded to group allocation of mice.

### Post-mortem human PD brain samples

Freshly frozen substantia nigra tissue blocks and cryostat sections from the brains of confirmed post-mortem human PD patients and age-matched neurologically normal individuals were procured from the brain banks at the Miller School of Medicine, University of Miami, FL, and the Banner Sun Health Research Institute, AZ. All post-mortem human samples were obtained, stored, and distributed according to the applicable regulations and guidelines involving consent, protection of human subjects and donor anonymity as described previously (32). For immunoblot experiments, tissue homogenates from freshly frozen tissue blocks were prepared at a final concentration of 1 mg/mL total protein from which 35 μg of total protein was directly used. Alternatively, total histone was extracted from tissue blocks and 15-45 μg of total histone was used.

### Immunoblotting

Cells were lysed with modified RIPA buffer, homogenized, sonicated, and finally centrifuged. The same procedure was utilized for processing animal tissues. Before being loaded onto Sodium Dodecyl Sulfate (SDS)-acrylamide gels, proteins were normalized based on their corresponding Bradford assay results. The standard curve utilized for this protein estimation was based on various serial dilutions of 1% BSA. In some cases, the total histone extracted was normalized by a Pierce BCA protein assay kit (Cat. #23225).

For protein separation, 20-40 μg of protein or 15-25 μg of total histone was loaded into the corresponding well of a 15% acrylamide gel. Gels were run at 103 V for 2.5 h at 4°C. Proteins were then transferred to a nitrocellulose membrane at 25 V overnight at 4°C. Following the transfer, membranes were blocked with LI-COR blocking buffer for 1 h and incubated in primary antibodies, according to the manufacturer’s protocol. Next, the membranes were washed with PBS-TWEEN 20 (0.05%) for 1 h and incubated in LI-COR IR secondary antibodies for 1 h on a Belly Dancer (Stovall Life Sciences, Greensboro, NC; setting 3, slow movement) at room temperature (RT). After washing with PBS-TWEEN 20 for 1 h, membranes were imaged by a LI-COR Odyssey scanner. The primary antibodies used were as follows: H3K27ac (Cell Signaling, 1:1000), p300 (Cell Signaling, 1:1000), iNOS (Santa Cruz, 1:750), H3 (Millipore, 1:1000) and β-actin (Sigma, 1:10000) as loading control. The secondary antibodies used were as follows: IR-800 conjugated goat anti-mouse IgG (LI-COR, 1:20000) and IR-700 conjugated goat anti-rabbit IgG (LI-COR, 1:20000).

### Immunocytochemistry and confocal imaging

The ICC protocol previously developed by Huang et al. (ref) was followed, in which the cells were first fixed with 4% paraformaldehyde (PFA), and then double-washed, blocked with blocking buffer, and incubated in primary antibodies. Following the primary antibody incubation (H3K27ac, 1:1000, Cell Signaling; p300, 1:1000, Cell Signaling; iNOS, 1:750, Santa Cruz; H3K4me1, 1:1000, Cell Signaling; H3K4me3, 1:1000, Cell Signaling), the Alexa dye-conjugated secondary antibody was added to the cells following the repeated PBS wash step. After the secondary incubation period finished, the cells were washed with filtered PBS and then left in Millipore water for approximately 1 min prior to being mounted. Cell coverslips were mounted onto microscope slides *via* Fluoromount aqueous mounting medium (Sigma) and then dried at RT while protected from light. The images were taken with an inverted fluorescence microscope Keyence BZ-X800 (Itasca, IL). Z-stack images of mitochondria were captured by confocal imaging. Quantification of fluorescence intensity was performed by automated BZ-X800 analyzer. For **Figure 2A**, cell body size enlarged upon treatment, therefore, automated BZ-X800 analyzer was set up to quantify the total fluorescence intensity of H3K27ac per cell rather than intensity peaks.

### MitoROS measurement

MitoROS generation was quantified using the MitoSOX red fluorescent indicator. MMCs were placed onto coverslips in a 24-well plate. After treatment, we added the MitoSOX probe (1 µM) to the cells. Fluorescence expressed by the generated mitochondrial superoxides was measured as per the manufacturer’s instructions.

### Mitochondrial membrane potential

The standard commercial protocol was followed for Invitrogen’s JC-1 mitochondrial potential sensor. In short, 2.0 μg/mL of JC-1 was diluted in 2% FBS-containing MMC media. This was then added to MMC cultures and incubated at 37°C for 20 min. Following this incubation, the cultures were gently triple-washed with PBS. Immediately after the washes, images were captured on the Keyence microscope before cells dried out. The ratio of red to green channel feedback was calculated based on the image analyzer’s results.

### Mitochondrial visualization and superoxide production

Following the standard commercial protocol for MitoTracker (Invitrogen), after the treatment paradigms described for cell culture treatments above, 300 μL of 166-nM CMXROS MitoTracker red dye diluted in 2% FBS-containing DMEM/F-12 medium was added to cell cultures and incubated at 37°C for 12 min. After gentle, triple washes with PBS, cells were fixed in 4% PFA for 30 min prior to following the steps for ICC and imaging.

### SYBR Green RT-qPCR

RNA was isolated from the treated cells through the utilization of TRIzol reagent following the manufacturer’s protocol. Essentially, the cells were first lysed in 0.5 mL of TRIzol reagent during a 5-min incubation at RT. Then 0.1 mL of chloroform was added to the sample and the tubes were inverted to mix. The samples were then incubated for another 2 min and then centrifuged. Following centrifugation, the top clear layer was placed into a new tube containing 0.35 mL isopropanol. Another 150-min incubation at RT followed this and then centrifugation was performed again. After removing the supernatant, the RNA pellets were washed with 75% ethanol, air-dried, and finally re-suspended in warm ultra-pure RNase-free water. The sample nucleic acid concentration was then measured through a NanoDrop spectrophotometer and all samples were normalized to the lowest concentration by the addition of ultra-pure RNase-free water. The samples were then processed *via* high-capacity cDNA synthesis (kit 4368814, Applied Biosystems, Waltham, MA) following the manufacturer’s protocol. For normalization of each sample in qPCR, the 18S rRNA gene primer set (Cat. No. PPM57735E, Qiagen, Germantown, MD) was used. Based on manufacturer’s guidelines, dissociation curves and melting curves were run to ensure that single amplicon peaks were obtained without any non-specific amplicons. Fold change in gene expression was determined using the ΔΔC_t_ threshold cycle (C_t_) method. Quantitative PCR was performed using RT^2^qPCR SYBR Green Master Mix (Agilent, Santa Clara, CA).

### Griess assay

Microglial cells were plated in 96-well plates and treated in 2% FBS-containing DMEM/F-12 medium. Following treatment, 50 μL of medium was collected, and incubated with 50 μL of Griess reagent for 10 min. A plate reader was utilized to read the absorbance at 540 nm. Sodium nitrite solution was used for making the standard curve.

### Histone extraction

According to manufacture protocol, cytosolic extraction reagent 1 provided by the NE-PER kit (Thermo Fisher Scientific) and 0.5% Triton X-100 were added to samples for 10 min. After incubation, the pelletized nuclei were collected following centrifugation at 2000 x g for 5 min and then resuspended in 0.2 N HCl for overnight incubation at 4°C for complete dissolution of histones. The following morning, samples were centrifuged at maximum speed for 10 min to collect pellets.

### Study approval

All animal studies followed animal procedures approved by ISU’s (Ames, IA, USA) Institutional Animal Care and Use Committee (IACUC). For human samples, since the post-mortem human brain tissues were obtained de-identified from approved national brain banks, Institutional Review Board (IRB) approval from ISU was not required.

### Statistical analysis

Data analysis was performed using GraphPad Prism 7.0. Normally distributed raw data were analyzed with either Student’s t-test (2-group comparisons) or one-way ANOVA (≥3-group comparisons) with Tukey *post hoc* test unless otherwise mentioned. Statistically significant differences were denoted as * p ≤ 0.05, ** p<0.01, and *** p<0.001.

## Discussions

**Figure 6:**
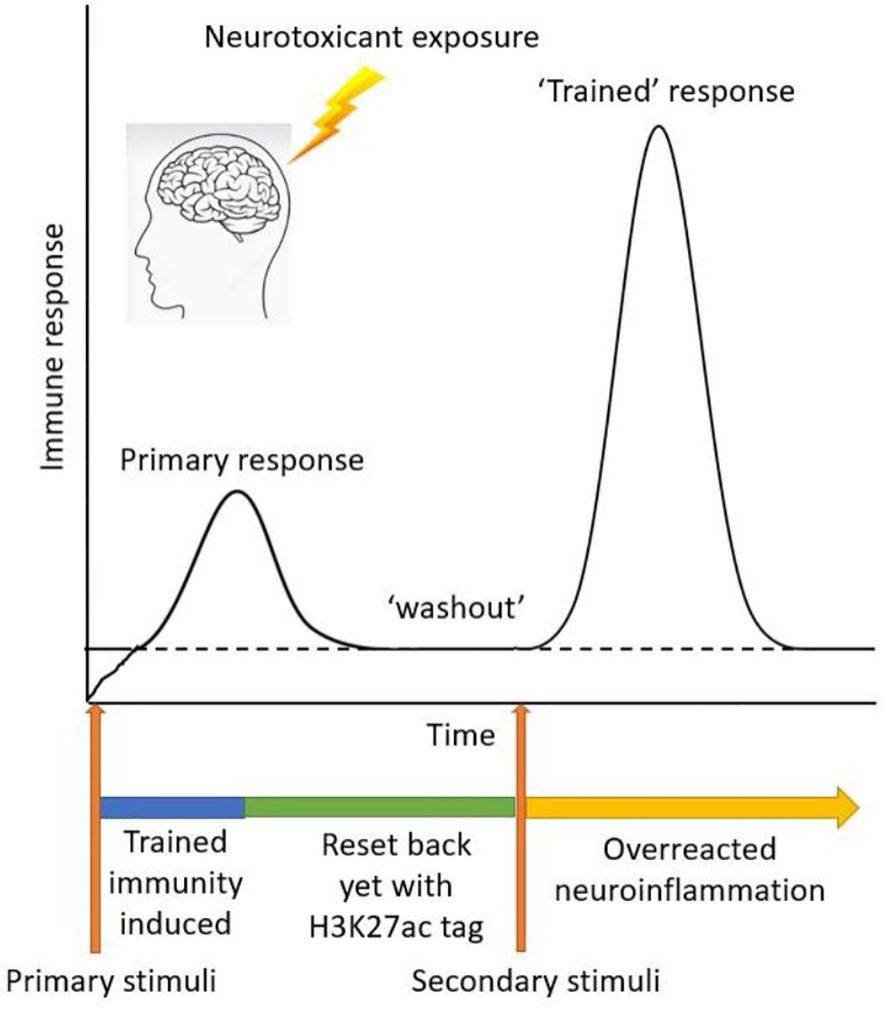
Schematic diagram of trained immunity treatment paradigm using LPS as a primary memory trigger and Mn as a secondary environmental stimulus.

In this study, we investigated the emerging concept of trained immunity as it relates to environmentally linked PD. Initial immune challenges can exacerbate subsequent insults because of the epigenetic reprogramming of histone modifications, which induce some changes in the epigenetic marks on the chromatin. This phenomenon of trained immunity leading to aberrant microglial activation would be an amplifier of PD and its progression. We here studied Mn exposure in an LPS or aggregated α-synuclein priming paradigm of trained immunity, in which microglia were given a certain amount of recovery time before the secondary insult with Mn. We provide evidence of similarities, in the context of environmentally linked PD, that trained immunity shares with microglial priming (**Figure 6**).

It is known that microglial priming followed immediately by a secondary stimulus induces large inflammatory responses, but we did not know if these primed states were either long-lasting or transient in their capacity to heighten inflammatory responses. Prior evidence from our group shows histone acetylation positively correlates with the pathogenesis of environmentally linked PD (19, 33). *In vivo* studies demonstrate that immune memory *via* epigenetic reprogramming shapes the hallmark pathology of neurodegenerative diseases (2, 14). Peripherally applying inflammatory stimuli in mouse brains sufficiently induces acute immune training and leads to differential epigenetic reprogramming of microglia that persists for at least six months (2). With regards to clinical relevance, evidence of epigenetic reprogramming and exaggerated inflammatory responses have been shown in disease patients. Heightened cytokine production and H3K4me3 deposition in the promoter region have been observed in circulating monocytes of patients with hypercholesterolemia (34). However, to the best of our knowledge, little or no evidence directly linked trained immunity to PD patients. We suspected that even after cessation of environmental toxicant exposure, long-lasting changes may be incorporated *via* epigenetic reprogramming of histone modifications. This study validated the important deposition of epigenetic marks *via* H3K27ac (along with H3K4me1 and H3K4me3) in microglia to shape long-term immune memory. We show evidence of increased deposition of these trained immunity memory marks (i.e., H3K27ac, H3K4me3, and H3K4me1) in the substantia nigra of human PD patients, indicating the clinical significance of epigenetic reprogramming. With a focus on H3K27ac, we applied a novel p300/H3K27ac pharmacological inhibitor to successfully suppress microglial memory formation. In addition, we identified iNOS signaling as the H3K27ac memory-associated inflammatory pathway.

The main driver behind trained immunity is the epigenetic modification of transcriptional pathways and metabolic reprogramming. Epigenetic reprogramming at the level of histone H3 modification has been proposed as the mechanism responsible for the long-lived memory of innate immunity (14). Trained immunity resembles the adaptive immune response in the way that it is associated with a heightened immune reaction that can be latently triggered in response to subsequent insults (15). However, unlike adaptive immune response, trained immunity has no specificity to antigen and the involvement of epigenetic reprogramming is different (6, 15). Immunotherapies already harness the ability of trained immunity to dramatically boost subsequent immune responses to target human diseases, e.g., vaccines through repeated stimuli (35) and cancer as personalized medicine (36). Recently, trained immunity has been explored in COVID-19 (37, 38). Despite its beneficial effects, uncontrolled and chronic trained immunity can be detrimental. Maintaining microglia in a prolonged hyperactive state increases the risk of developing Parkinsonism or PD. Functional changes (e.g., excessive inflammation and tissue damage in the brain) and transcriptional alteration induced by epigenetic remodeling have been implicated to play a significant role in the maintenance of PD (6, 39).

The process of accelerating the innate response by priming the same innate receptors suggests that trained immunity might employ a similar mechanism with microglial priming (40), and others have even equated the processes (3, 6, 14). Additionally, environmental exposure-induced priming activates microglia and renders them susceptible to secondary stimuli, triggering heightened cytokine production similar to trained immunity. The evidence in our priming paradigm shows the similarities shared between trained immunity in this study and a previous microglial priming study (8), including mounting levels of inflammatory responses (e.g., cytokines release, NLRP3 upregulation, and IL-1β secretion in post-priming and post-recovery), iNOS signaling, and reactive oxygen species (ROS). We know that in both conditions, microglia stay in a primed or “alerted” state after the initial stimulus and can accrue increased sensitivity over time (3, 7, 14). Although the mechanisms underlying microglial priming and trained immunity have not yet completely defined, one distinct difference between them is that trained immunity has two forms – ‘trained’ and ‘desensitized.’ Regarding the use of ‘trained’ microglia in this study, initial triggers ‘prime’ microglia and induce excessive responses to secondary or subsequent stimuli (3, 7, 40). In contrast, microglia may be ‘desensitized’ by multiple insults to the point of becoming ‘immune tolerant’ (1–3).

The ‘trained’ phenomenon of trained immunity mirrors that of B cells leading to antigen-specific CD4^+^ T cell expansion, memory formation and cytokine production (41). In B cells, a delicate regulatory network is required to achieve the optimal signal output of immune responses as do microglia. The viral infection could cause the activation of the lymphocytes, and the autoantigen could maintain this activation even after the eradication of the infectious agent, in line with our findings of trained microglia. An increasing incidence of autoimmunity can exacerbate the inflammatory process and lead to sustained inflammation and enhanced responses (42). Immunological memory against reinfection by past experiences is formed (43). During recall responses, memory B cells respond by proliferating effector cytokines at a faster rate (43). Epigenetic changes regulate B cell activation and differentiation (44). Unlike trained immunity, the remembrance of exposure to previous encounters in the adaptive immune system with B cells is antigen-specific and delayed (45).

As the underlying mechanism of trained immunity, epigenetic reprogramming holds the labile nature originating from rapid deacetylation kinetics. This nature renders its translational potential as a target for PD treatment (46, 47). Changes in epigenetic marks may not always alter global, distinct transcriptional or translational expression in microglia, but such changes might modify certain important factors in the pathways of neuroinflammation. H3K27ac is one of the important epigenetic marks in trained immunity (48). In this study, iNOS expression was found to be regulated by H3K27ac. But some other inflammatory pathways may also be regulated by H3K27ac. In some other cases, multiple epigenetic marks may work together to remodel microglial function. Currently, it is not well-studied which marks tend to dictate the role of epigenetic regulation nor do we know how double, triple, and even quadruple marks work. The potential challenge in exploring this question is that the field lacks epigenomic technology to precisely target the specific combination of epigenetic marks. Additionally, considering the complex cell-to-cell interactions in the brain, it is not clear how cells might affect the epigenetic marks in other cells. Furthermore, the question of how repeated exposure to environmental insults triggers an accumulation of epigenetic modifications at multiple times (i.e., increasing the number of epigenetic changes) and locks microglia in a hyperactive state is very important to study.

As a key transcriptional co-activator essential for cellular processes, p300 catalyzes the acetylation of H3K27 specifically at enhancer regions. Regarding the transcriptional effects of p300 in inflammatory responses, little has been reported. An *in vitro* study in microglia revealed that p300 mediates anti-inflammatory effects by upregulating the Nrf2-p300 pathway and downregulating p65-p300 signaling upon curcumin treatment (49). It is reported that p300 regulates GATA4 in cardiogenesis by mediating H3K27ac (50). In cancer malignancy, USP24 stabilizes p300, increases the levels of H3 acetylation and NF-κB, and induces IL-6 (51). Due to its feasibility of selectively targeting the catalytic activity of histone acetyltransferases and its significance in cellular processes, p300 has become an attractive target for epigenetics-based cancer treatment (52). Nevertheless, its effects on regulating microglial inflammation (especially immune memory formation) in the context of neurodegenerative disease has not been well studied. It has been proven that p300 plays a critical role in controlling mitochondrial function and its stress response (53, 54). Recently, the p300 inhibitor GNE-049 is suggested to have translational potential in PD treatment (55). GNE-049 is a highly potent and selective bromodomain inhibitor of CBP/p300 (55). This inhibitor selectively blocks H3K27ac at enhancers (21, 56), while having no effect on H3K18ac levels (57). For this reason, it has been used as a tool to investigate the impact of H3K27ac inhibition (21). As a possible link between histone acetylation and mitochondrial function in neuroinflammation (8), our results show that GNE-049 reduces mitochondrial stress and the neuroinflammatory response.

Overall, our data, in exploring the novel concept of trained immunity, show that immune challenges shaped microglial memory *via* deposition of histone modifications. The study broadens our understanding of the triggers and modulators of neuroinflammation, thereby identifying a potential avenue for dampening the risks posed by exposure to environmental toxicants. Systematic testing GNE-049 or related compounds for pharmacological inhibition and translational potential in PD might provide a novel therapeutic strategy. Quantifying epigenetic deposition in correlation with the severity of PD may lead to exciting advances in our understanding of the epigenetic regulation of PD. Furthermore, to gain a better understanding of the mechanism underlying trained immunity, efforts are needed to establish more treatment paradigm options and mouse models developed specifically for trained immunity research.

## Data availability statement

The raw data supporting the conclusions of this article will be made available by the authors, without undue reservation.

## Ethics statement

Ethical review and approval was not required for the study on human participants in accordance with the local legislation and institutional requirements. Written informed consent for participation was not required for this study in accordance with the national legislation and the institutional requirements. The animal study was reviewed and approved by ISU’s (Ames, IA, USA) Institutional Animal Care and Use Committee (IACUC). Written informed consent was not obtained from the individual(s) for the publication of any potentially identifiable images or data included in this article.

## Author contributions

MH and AGK conceived this project and supervised the experiments. MH and AGK designed research. MH and AE performed experiments. MH, AE and EM analyzed experimental results. MH, EM, HJ, VA, AK, and AGK prepared the manuscript. All authors contributed to the article and approved the submitted version.
