# Supplementary material for: Microglial immune regulation by epigenetic reprogramming through histone H3K27 acetylation in neuroinflammation
Source: Front Immunol. 2023 Mar 22;14:1052925. doi: 10.3389/fimmu.2023.1052925 (PMC10073546; doi:10.3389/fimmu.2023.1052925)
Supplement: Supplementary file 1 [file DataSheet_1.docx]

**Supplementary Materials**

Microglial Immune Regulation by Epigenetic Reprogramming Through Histone H3K27 Acetylation in Neuroinflammation

**Authors**

Minhong Huang^1^, Emir Malovic^1^, Alyssa Ealy^1,2^, Huajun Jin^1,2^, Vellareddy Anantharam^1,2^, Arthi Kanthasamy^1,2^, Anumantha G. Kanthasamy^1,2*^

**Affiliations**

^1^Parkinson Disorders Research Laboratory, Iowa Center for Advanced Neurotoxicology, Department of Biomedical Sciences, 2062 Veterinary Medicine Building, Iowa State University, Ames, IA 50011

^2^Center for Neurological Disease Research, Department of Physiology and Pharmacology, 325 Riverbend Road, University of Georgia, Athens, GA 30602

***Correspondence:** Anumantha Kanthasamy, Ph.D., Professor, Johnny Isakson Chair, Georgia Research Alliance Eminent Scholar, and Director, Center for Neurological Disease Research, Department of Physiology and Pharmacology, 325 Riverbend Road, Center for Molecular Medicine Bldg, University of Georgia, Athens, GA 30602. Telephone: (706) 542-2380; Fax: (706) 542-4412; Email: anumantha.kanthasamy@uga.edu


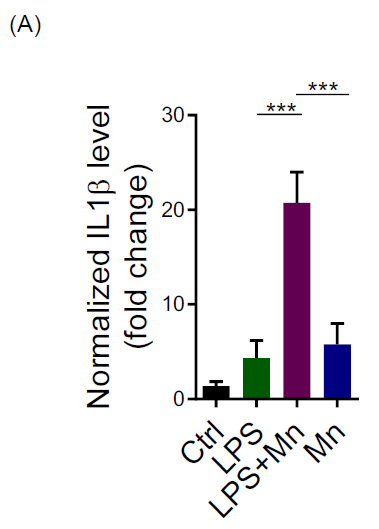


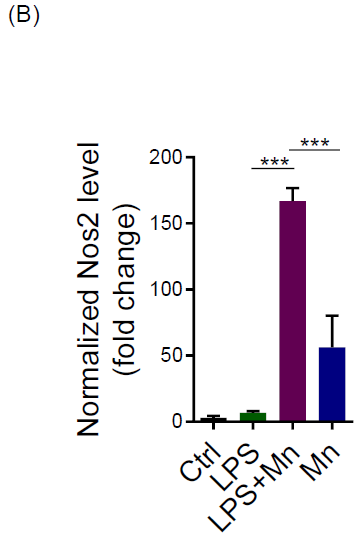


**Supplementary Figure 1.** **Enhanced inflammatory responses upon secondary stimuli after 48-hour recovery.** RT-qPCR analysis of (A) *IL-1β* and (B) *Nos2* in primed MMCs with 48-h recovery. Two individual experiments (n=5~6) were performed. Data show mean ± SEM of one-way ANOVA followed by Tukey’s post hoc test. Ctrl, control; ***p<0.001.


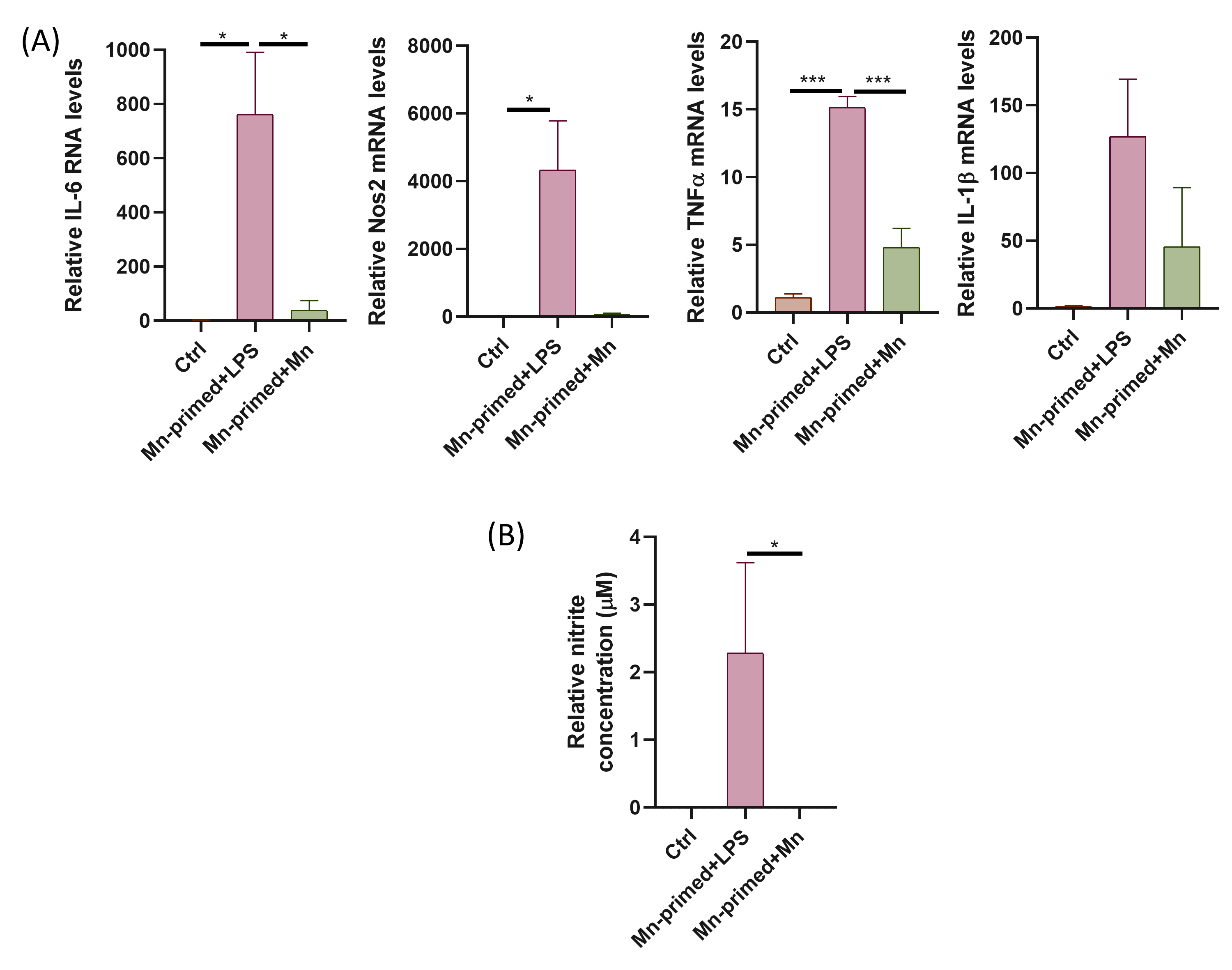


**Supplementary Figure 2. Mn priming can induce trained immunity with LPS being a more potent second insult as compared to Mn.** (A) RT-qPCR analysis from Mn-primed MMCs with a secondary insult of either Mn 100 μM or LPS 1 μg/mL shows pro-inflammatory cytokines levels with *IL-6*, *Nos2*, *Tnfα*, and *IL-β*. (B) Nitrite levels from Mn-primed MMCs with a secondary insult of either Mn 100 μM or LPS 1 μg/mL. Data show mean ± SEM of one-way ANOVA followed by Tukey’s post hoc test. Ctrl, control; *p≤0.05 and ***p<0.001.

**
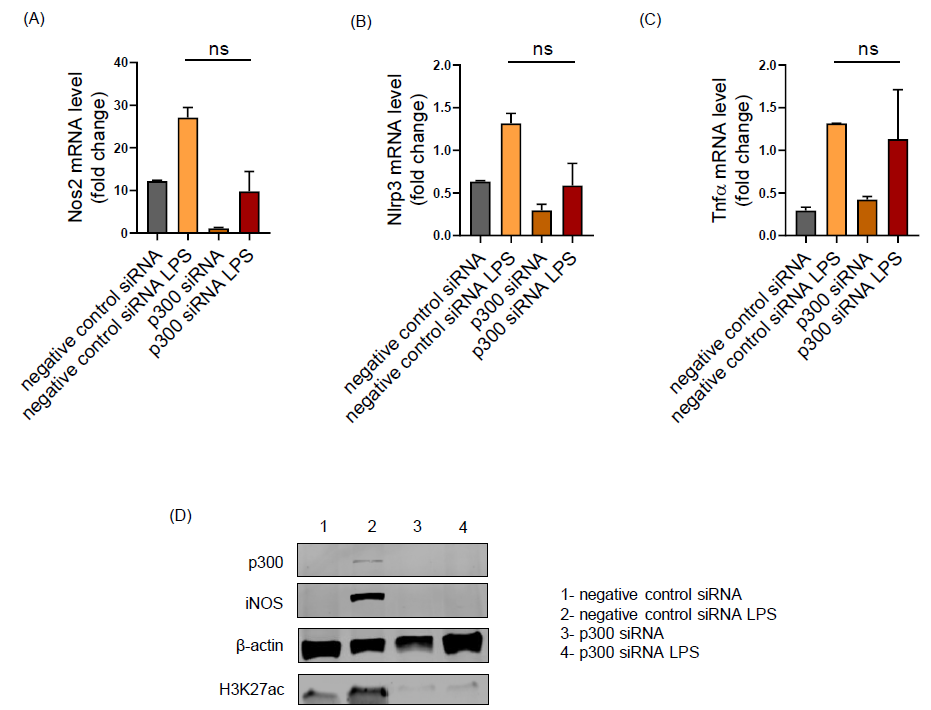
**

**Supplementary Figure 3. p300 knockdown decreases the transcription expression of LPS-induced inflammatory factors**. RT-qPCR analysis shows the relative expression levels of *Nos2* (A), *NLRP3* (B), and *Tnfα* (C) in p300 KD upon LPS treatment, compared to MMCs with negative control siRNA. (D) Western blot of p300, iNOS, and H3K27ac in p300 KD MMCs. Data show mean ± SEM of one-way ANOVA followed by Tukey’s post hoc test. ns, not significant.

**
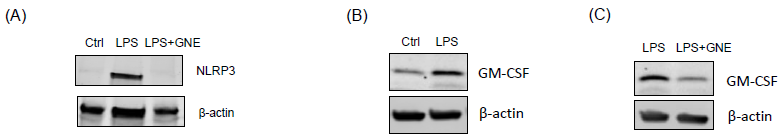
**

**Supplementary Figure 4.** **Translation levels of inflammation-associated factors upon GNE-049 inhibition of immune regulation.** Immunoblotting analysis of (A) NLRP3 and (B-C) GM-CSF in GNE-049 cotreated, trained MMCs.

**
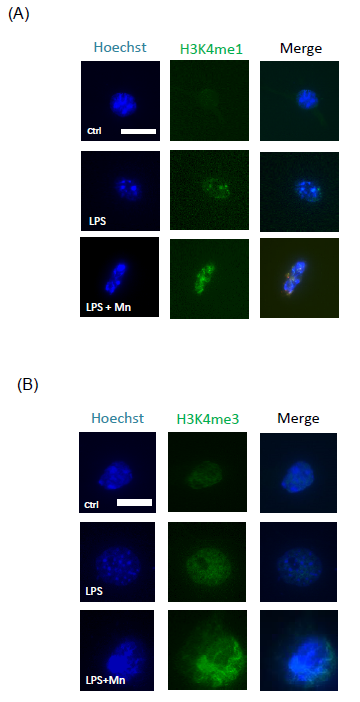
**

**Supplementary Figure 5.** **Increased H3K4me1 and H3K4me3 deposition in Mn-exposed, primed PMGs.** Immunofluorescence microscopy analysis of (A) H3K4me1 and (B) H3K4me3 in Mn-exposed, LPS-primed PMGs. Single PMG cell is shown because of their lower cell density resulting from their reduced viability during prolonged treatments. Scale bar, 10 µm.

**
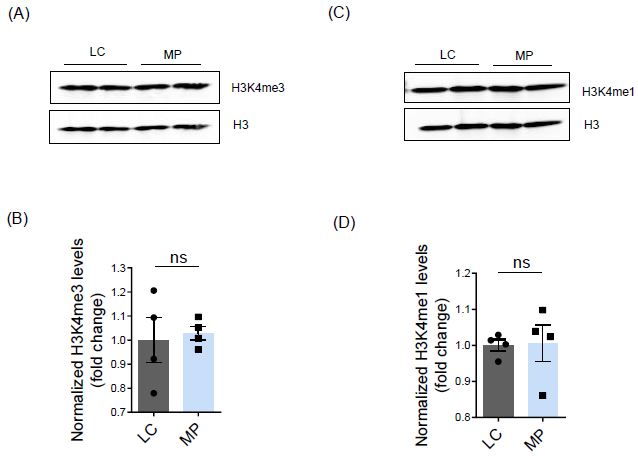
**

**Supplementary Figure 6.** **No significant change of H3K4me3 and H3K4me1 in olfactory bulbs of MitoParks.** Representative blots of H3K4me3 (A-B) and H3K4me1 (C-D) deposition with their respective quantification in the olfactory bulbs of MitoParks. MP, MitoParks; LC, littermate controls.
